# The Long-Term Effects of Non-Pharmacological Interventions on Diabetes and Chronic Complication Outcomes in Patients With Hyperglycemia: A Systematic Review and Meta-Analysis

**DOI:** 10.3389/fendo.2022.838224

**Published:** 2022-03-18

**Authors:** Rongrong Zhou, Yashan Cui, Yuehong Zhang, Jin De, Xuedong An, Yingying Duan, Yuqing Zhang, Xiaomin Kang, Fengmei Lian

**Affiliations:** ^1^ Guang’anmen Hospital, China Academy of Chinese Medical Sciences, Beijing, China; ^2^ Xiyuan Hospital, China Academy of Chinese Medical Sciences, Beijing, China; ^3^ Beijing University of Chinese Medicine, Beijing, China

**Keywords:** randomized controlled trial, meta-analysis, diabetes incidence, long-term, complications, non-pharmacological intervention

## Abstract

**Objective:**

This study aimed at examining the long-term effects of non-pharmacological interventions on reducing the diabetes incidence among patients with prediabetes and chronic complications events among patients with hyperglycemia (pre-diabetes and diabetes) by performing a systematic review and meta-analysis of randomized controlled trials (RCTs).

**Methods:**

PubMed, MEDLINE, EMBASE, the Cochrane Library, and the Web of Science Core Collection were searched for studies published between January 1990 and November 2021, looking for RCTs to evaluate the effects of non-pharmacological interventions on preventing the incidence of diabetes and chronic complications in comparison with medical therapy, placebo, or usual diabetes care. Two independent reviews extracted relevant data and quality assessment. Any discrepancies were resolved by a third reviewer.

**Results:**

In total, 20 articles involved 16 RCTs (follow-up ranged from 2 to 30 years) were included. Pooled analysis of intervention studies demonstrated clearly that non-pharmacological interventions have a significant effect on reducing the diabetes events in patients with prediabetes (RR 0.62; 95% CI 0.54, 0.71). Pooled analysis of extended follow-up studies showed that non-pharmacological interventions could effectively reduce the diabetes incidence in patients with prediabetes (RR 0.78; 95% CI 0.63, 0.96). Meta-regression and subgroup analysis indicates that the diabetes incidence of the long-term group (duration > 3 years) was clearly reduced by 0.05% compared with the relatively short-term group (duration ≤ 3 years). The incidence of microvascular complications in patients with hyperglycemia was effectively lowered by non-pharmacological interventions (RR 0.60; 95% CI 0.43, 0.83).

**Conclusion:**

Non-pharmacological interventions have a long-term effect on reducing the diabetes incidence among prediabetic patients and effectively preventing microvascular complications on hyperglycemia.

**Systematic Review Registration:**

https://www.crd.york.ac.uk/prospero/.

## Introduction

The condition of hyperglycemia is one of the most common chronic metabolic disorders, including prediabetes and diabetes. Prediabetic state is a high-risk group of diabetes and a potential risk factor for diabetes and cardiovascular disease, and impaired glucose tolerance (IGT) is more common than impaired fasting glucose (IFG) (1). According to the American Diabetes Association (ADA) expert group, up to 70% of patients with prediabetes will eventually develop diabetes (2). Without effective intervention, it will be more likely to worsen and develop into diabetes mellitus with macro- and microvascular conditions (3, 4). Diabetes complications, particularly macro- and microvascular diseases, are the leading cause of reduction in the quality of life of patients and increase in diabetic mortality. Previously large-scale meta-analyses have indicated that prediabetes is associated with an increased risk of cardiovascular diseases (5–8). Therefore, prevention of diabetes and its severe complications is urgently needed.

Currently, a growing number of clinics and patients pay more attention to non-pharmacological strategies due to the hypoglycemic agents having a limited role in the progression of diabetes and its complications (9). Different non-pharmacological strategies have been reported including lifestyle change, dietary modification, physical activity, and exercise with different intensity, with favorable and unfavorable records on diabetes and its complication prevention and development (10–12). Several large RCTs with a long-term follow-up such as Diabetes Prevention Program (DPP), the Finnish Diabetes Prevention Study, and Da Qing Diabetes Prevention have been reported, stating that adopting a healthy lifestyle would obtain long-term effects on preventing diabetes and its complications (13–15). Moreover, many recent systematic reviews have emphasized the important role in glycemic control and diabetes incidence (16–18). However, these systematic reviews only evaluated the effects on diabetes events among intervention studies, without assessing the effects of extended follow-up studies of intervention completed. In addition, few systematic reviews have reported the important role of non-pharmacological strategies in microvascular complications.

Therefore, it might be beneficial to conduct a systematic review and meta-analysis to whether comprehensive non-pharmacological interventions would be long-term effectiveness for preventing the diabetes and diabetes-related complications compared to other proposed treatments. The objective of this study, obtained by a comprehensive systematic review and meta-analysis of randomized controlled trials (RCTs), was to evaluate the long-term effects of non-pharmacological interventions on reducing the diabetes incidence in prediabetic patients and microvascular complications in patients with pre-diabetes and diabetes.

## Materials and Methods

This systematic review and meta-analysis was performed according to the Cochrane Handbook for Systematic Reviews of Interventions (https://gdt.gradepro.org/app/handbook/handbook.html). Data were reported in accordance with the Preferred Reporting Items for Systematic Reviews and Meta-Analysis (PRISMA) Guidelines (19). The PRISMA checklist of this study is provided in **Supplementary Table S1**. The study was registered in the International prospective register of systematic reviews (CRD42021240826).

### Data Sources and Search Strategy

The five databases including PubMed, MEDLINE, EMBASE, the Cochrane Library, and the Web of Science Core Collection were searched for eligible trials using the keywords “diabetes” or “hyperglycemia” or “Impaired glucose tolerance”; “Lifestyle intervention” or “physical activity” or “exercise”; “Macrovascular complications” or “Microvascular complications” or “diabetic nephropathy” or “Diabetic retinopathy” or “Diabetic peripheral neuropathy” or “Diabetic foot”; “diabetes incidence”. The initial search was performed in March 2021, and an updated search of five databases was performed in November 2021 using the same search terms. The search strategy for this study is provided in **Supplementary Table S2**.

### Study Selection

To be included, studies had to be RCTs, which made direct comparisons of non-pharmacological interventions with medical therapy, placebo, usual care, or standard care; included RCTs had at least a 2-year duration of intervention; patients should be of any age with hyperglycemia (i.e., type 1 diabetes mellitus, type 2 diabetes mellitus, prediabetes, impaired glucose tolerance, impaired fasting blood glucose) and without chronic complications of diabetes; included RCTs reported at least one of the main outcomes of interest (i.e., diabetes incidence, cardiovascular complications, microvascular complications); and for the articles in the same study, the article with the longest follow-up duration was included. We excluded studies where both the intervention group and the control group were non-pharmacological interventions, non-RCTs, and publications without original data or with incomplete data. A second author (YC) confirmed that all articles that met the inclusion and exclusion criteria were included in the meta-analysis.

### Data Extraction

Two independent investigators (RZ and (YC) used a standardized form to extract data from RCTs that met the inclusion/exclusion criteria, and any discrepancies were resolved by a third author (FL). The following relevant data from each article were extracted, including authors and year of publication; country; study design; study intervention and follow-up duration; patients’ data including type of hyperglycemia; intervention measures of treatment group and control group; diagnostic criteria of outcomes; and outcomes of diabetes incidence and chronic complications. When the outcomes were reported as the percentage of incidence, a conversion in number of diabetes and chronic complications has been made in order to conduct an analysis.

### Data Synthesis and Analysis

The primary outcomes of this meta-analysis were the diabetes incidence among prediabetic patients and microvascular events in patients with hyperglycemia. The meta-analysis was carried out using Review Manager 5.4 (The Cochrane Collaboration, The Nordic Cochrane Centre, Copenhagen, Denmark).

Meta-analysis was performed using a random-effect model because of possible clinical heterogeneity. Data were pooled into relative risks (RRs) for dichotomous outcomes and RCTs with 95% CI. Heterogeneity was assessed using the *I* ^2^ statistics, where *I* ^2^<30% was considered as low heterogeneity, *I* ^2^ values of 30–70 were considered as moderate heterogeneity, and *I* ^2^>70% was considered as high heterogeneity.

Sensitivity analyses for the study quality and certain study characteristics (i.e., intervention duration, follow-up duration, and type of treatment measures) were performed through removing each individual trial. After recalculating pooled-effect estimates and heterogeneity, changing the significance of the effect or altering the effect size by 10% or more was considered influential. The meta-regression and subgroup analysis was used to examine the effect of follow-up duration on heterogeneity between the studies. We assessed publication bias by visual inspection of funnel plots for any outcomes>10 articles.

### Quality Assessment

The Cochrane risk-of-bias tool was used to assess the RCT risk of bias. We assessed risk of bias in random sequence generation and allocation concealment, blinding of participants and personnel, blinding of outcome assessment, incomplete outcome data, and selective reporting. A sensitivity analysis was conducted to exclude the articles with relevant weaknesses in trial design or execution. The overall quality of the evidence was also assessed using Grading of Recommendations Assessment, Development and Evaluation (GRADE) working group guidelines (20). The quality of each RCT was assessed blinding by two reviews (RZ and (YC), and disagreements were resolved by a third author (FL).

## Results

### Literature Search

**Figure 1:**
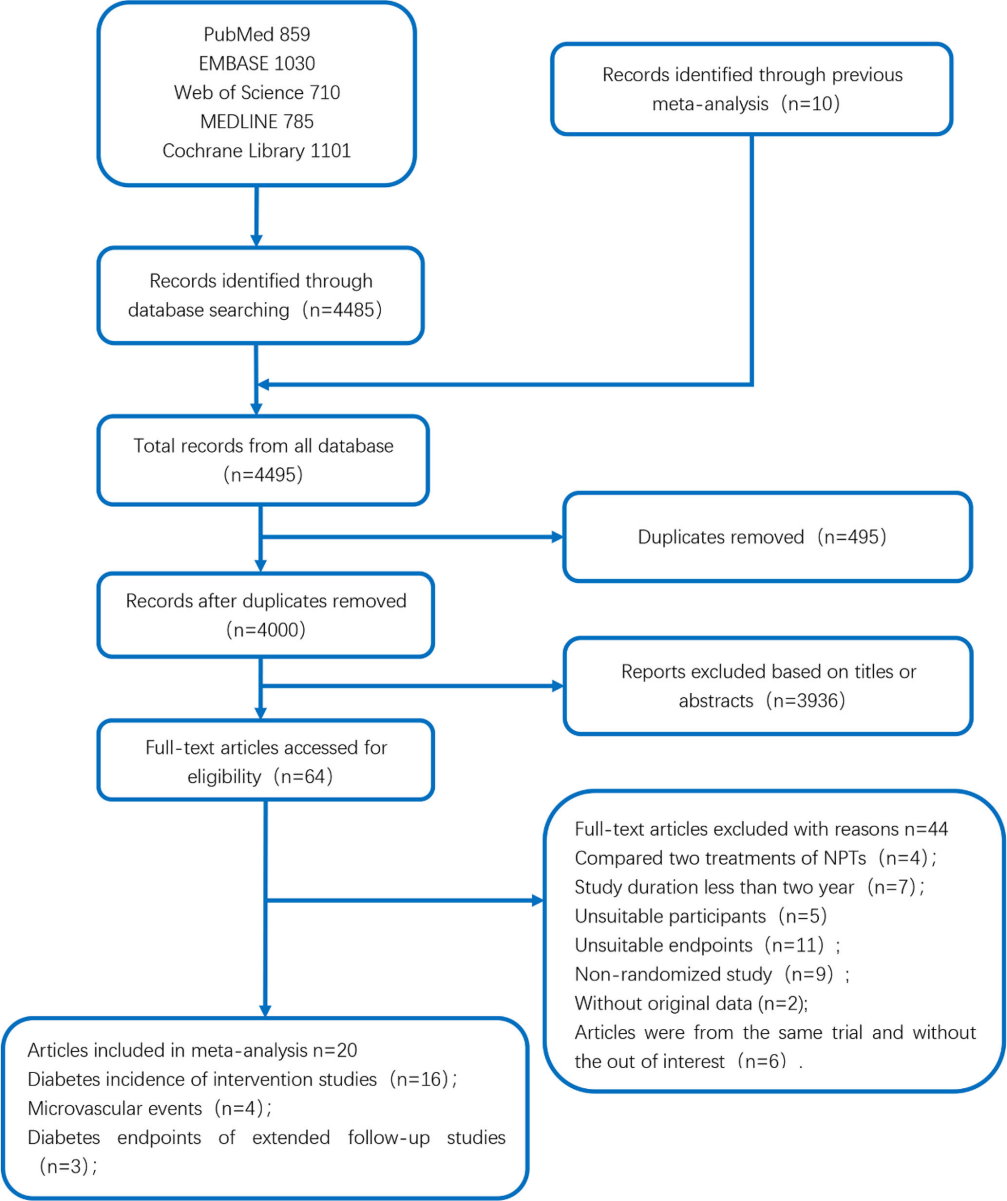
The flow diagram of search and selection process used for studies included in the meta-analysis.

The initial search assessed 4,495 publications. After the removal of duplicates, a total of 4,000 studies were selected and 3,936 publications were excluded by screening the titles and abstracts. Of the remaining 64 publications, 44 were excluded (**Supplementary Table S3**) because of study duration less than 2 years (7 publications), comparison of 2 treatments of non-pharmacological interventions (4 publications), unsuitable endpoints (11 publications), non-randomized study (9 publications), without original data (2 publication), unsuitable participants (5 publications), and articles being from the same trial and without the outcome of interest (6 publications). Finally, 20 suitable publications were included in the quantitative synthesis meta-analysis (**Figure 1**).

### Study Characteristics

The main characteristics of these eligible articles are shown in **Table 1**. We identified 20 articles (14, 21, 23–30, 32–38) and 16 RCTs used for the main analysis. The sample size ranged from 74 to 2,161. The length of total duration across all trials ranged from 2 to 20 years. The length of intervention ranged from 2 to 15 years, and the length of the extended follow-up ranged from 0 to 14 years. The 15 articles (21, 23, 25, 26, 28, 29, 32–39) reported the outcome of diabetes events among intervention studies, and the 3 articles (22, 24, 25) reported the outcome of diabetes events among extended follow-up studies of intervention completed. The 4 articles (14, 22, 27, 30) reported the outcome of microvascular complications. In these four articles, one article (30) reported the study conducted in patients with diabetes, the rest of which were conducted in prediabetic patients. The two articles (21, 22) reported on the Da Qing Diabetes Prevention Study, two (23, 24) on the Indian Prevention Study, and two (26, 27) on the American Diabetes Prevention Program. The primary outcome was change in the number of diabetes events in 15 articles and 3 extended follow-up studies, and microvascular complications in 4 articles.

**Table 1:** The main characteristics of eligible articles included in the meta-analysis.

Study	First author (year)	Country	Participants	I (number)	C (number)	Outcomes of I	Outcomes of C	Duration of intervention	Duration of extended follow-up	Total length	Diagnostic criteria
**The DaQing Diabetes Prevention Study**	Pan (1997) (21)	China	IGT	Lifestyle intervention (diet and exercise) (397)	Standard care (133)	52 T2DM	30 T2DM	6 years	0 year	6 years	WHO 1985
	Gong (2011) (22)			Lifestyle intervention (409)	Standard care (133)	309 DM; 66 MVD	118 DM; 26 MVD	6 years	14 years	20 years	
**The Indian Diabetes Prevention Study**	Ranchamdran (2013) (23)	India	IGT	Mobile phone messaging of lifestyle modification (271)	Standard care (266)	50 T2DM	73 T2DM	2 years	0 year	2 years	WHO 1999
	Nanditha (2018) (24)			Mobile phone messaging of lifestyle modification (183)	Standard care (163)	29 DM	33 DM	2 years	3 years	5 years	
**The Finnish Diabetes Prevention Study (DPS)**	Lindström (2006) (25)	Finnish	IGT	Lifestyle 265	Standard Care 257	44 DM	76 DM	4 years	0 year	4 years	WHO 1985
				Lifestyle 238	Standard Care 237	75 DM	110 DM	4 years	3 years	7 years	
	Aro (2019) (14)			Lifestyle intervention (113)	Control group (98)	27 MVD	37 MVD	4 years	5 years	9 years	
**The American Diabetes Prevention Program (DPP)**	Knowler (2002) (26)	American	PDM	Intensive lifestyle intervention (1079)	Placebo (1082)	155 DM	313 DM	2.8 years	0 year	2.8 years	ADA
	The Diabetes Prevention Program Research Group (2015) (27)			Lifestyle intervention (751)	Metformin with placebo (1552)	103 MVD	236 MVD	10 years	0 year	10 years	
**The European Diabetes Prevention Study**	Penn (2009) (28)	The UK	IGT	Individual motivational interviewing (39)	Usual care (38)	5 T2DM	11 T2DM	3.1 years	0 year	3.1 years	WHO 1999
**Study on Lifestyle Intervention and Impaired Glucose Tolerance Maastricht (SLIM)**	Roumen (2008) (28)	Netherlands	IGT	Lifestyle intervention (44)	Usual care (47)	8 T2DM	18 T2DM	3 years	0 year	3 years	WHO 1999
**The Let’s Prevent Diabetes Cluster Study**	Davies (2016) (29)	The UK	PDM	Lifestyle intervention (356)	Standard care (360)	64 T2DM	67 T2DM	3 years	0 year	3 years	WHO 1990
**The Exercise Training DPN Prevention Study**	Balducci (2006) (30)	Italy	DM	Exercise training (31)	Sedentary lifestyle (47)	4 DPN	10 DPN	4 years	0 year	4 years	Feldman et al., 1994
**The Japanese Diabetes Prevention Study**	Kosaka (2005) (31)	Japan	IGT	Lifestyle 102	Standard Care 356	3 DM	33 DM	4 years	0 years	4 years	WHO1980
**The Indian Diabetes Prevention Programme**	Ranchamdran (2006) (32)	India	IGT	Lifestyle intervention (120)	Control group (382)	47 T2DM	173 T2DM	3 years	0 year	3 years	WHO 1999
**Liao et al.**	Liao (2002) (33)	USA	IGT	Lifestyle (36)	Control (38)	1 DM	2 DM	2 years	0 year	2 years	WHO 1985
**Lindahl et al.**	Lindahl (2009) (34)	Sweden	IGT	Lifestyle (83)	Usual care (85)	17 DM	23 DM	5 years	0 year	5 years	WHO 1985
**Saito et al.**	Saito (2011) (35)	Japan	IFG	Lifestyle (311)	Control (330)	35 DM	51 DM	3 years	0 year	3 years	WHO 1999
**Sakane et al.**	Sakane (2011) (36)	Japan	IGT	Lifestyle (152)	Control (152)	9 DM	18 DM	3 years	0 year	3 years	WHO 1985
**Zong et al.**	Zong (2015) (37)	China	PDM	Nutrition (107)	Control (107)	3 DM	11 DM	2 years	0 year	2 years	WHO 1985
**Hellgren et al.**	Hellgren (2016) (38)	Sweden	PDM	Physical activity (66)	Usual care (30)	10 DM	7 DM	3 years	0 year	3 years	IDF 2011

I, Intervention group; C, Control group; ADA, American Diabetes Association; WHO, World Health Organization; DPP, The American Diabetes Prevention Study; DPS, The Finnish Diabetes Prevention Study; SLIM, The Maastricht Diabetes Prevention Study; IGT, impaired glucose tolerance; DM, diabetes mellitus; T2DM, type 2 diabetes mellitus; DR, diabetic retinopathy; DN, diabetic nephropathy; DPN, diabetic peripheral neuropathy; PD, pre-diabetes mellitus; CVD, cardiovascular disease; MVD, microvascular disease.

### Long-Term Effects on the Prevention of Diabetes in Intervention Studies

**Figure 2:**
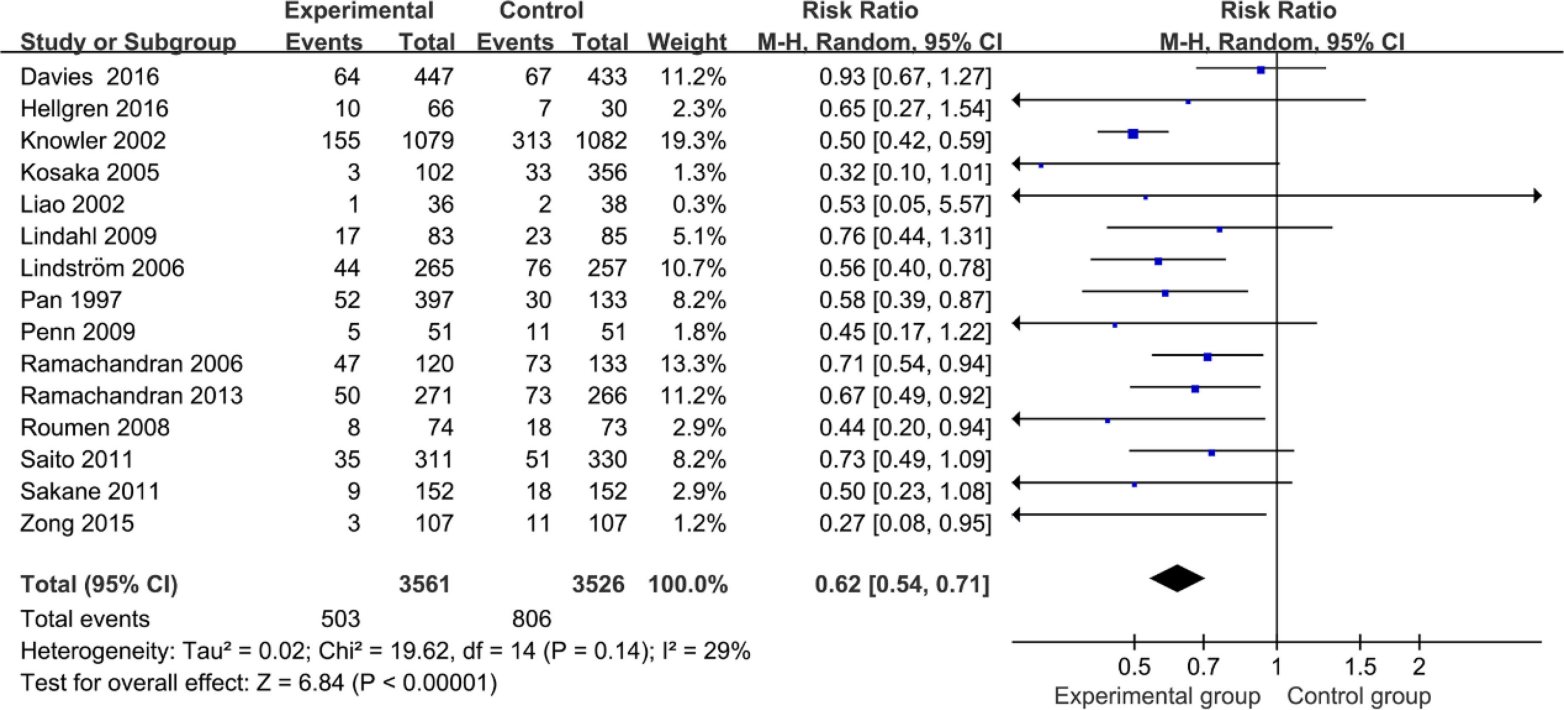
Forest plot of diabetes incidence in intervention studies.

This analysis selected the results of the study that reported the longest follow-up time of diabetes events in terms of the same RCT. In overall analysis of 15 studies, as **Figure 2** shows, the non-pharmacological interventions led to a diabetes incidence decrease significantly greater than those of comparator groups (0.62; 95% CI 0.54 to 0.71, *p* < 0.00001), with low heterogeneity between studies (*I* ^2^ = 29%, *p* = 0.14).

### Effects on Microvascular Complications

**Figure 3:**
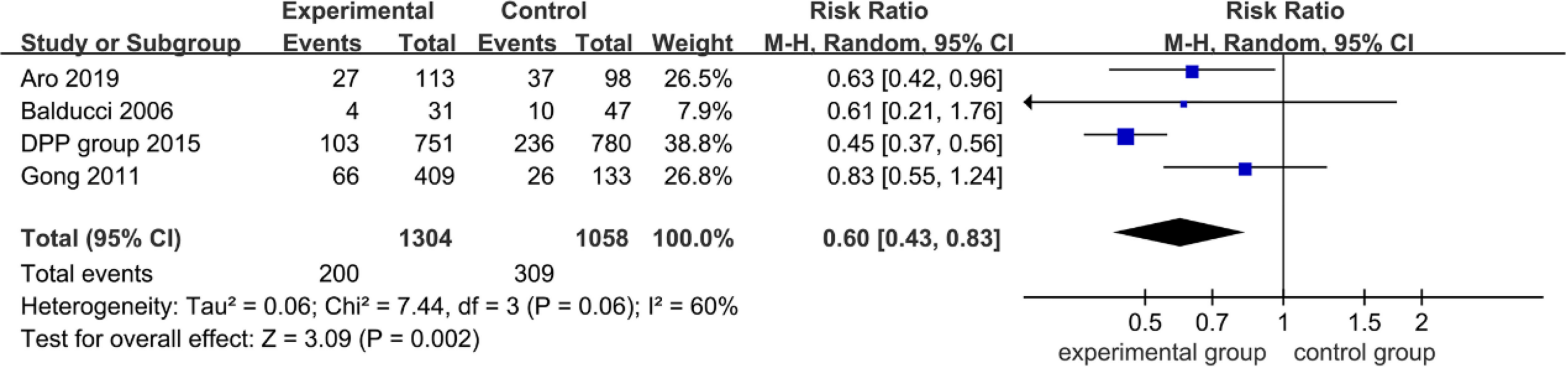
Forest plot of microvascular events.

**Figure 3** shows the long-term effect on lowering microvascular events among patients with hyperglycemia. Compared with usual care and medical treatment, those with non-pharmacological interventions among hyperglycemic individuals had a lower incidence of microvascular complications (0.60; 95% CI 0.43 to 0.83, *p* = 0.002); heterogeneity across articles was moderate with an *I* ^2^ of 60% (*p* = 0.06). In sensitivity analysis, the heterogeneity of combined estimates did mark a change with the exclusion of Gong et al. or the DPP group (**Table 2**), indicating that the source of heterogeneity may be due to the great variation in the duration of follow-up (follow-up duration of Gong et al. was 20 years, and that of the DPP group was 15 years).

**Table 2:** The sensitivity analysis outcomes of microvascular events.

Removal of study	Intervention, N	Control, N	Pooled effect estimate			Heterogeneity	
			RR	95% Cl	p-value	I^2^	p_Z_
None	0	0	0.60	[0.43, 0.83]	0.06	60%	0.002
Aro, 2019 (14)	27/113	37/98	0.59	[0.37, 0.95]	0.04	70%	0.03
Balducci, 2006 (30)	4/31	10/47	0.60	[0.41, 0.87]	0.02	73%	0.008
Gong, 2011 (22)	66/409	26/133	0.49	[0.40, 0.61]	0.34	7%	0.0001
The Diabetes Prevention ProgramResearch Group, 2015 (27)	103/751	236/780	0.72	[0.54, 0.95]	0.64	0%	0.02

### Long-Term Effects on the Prevention of Diabetes in Extended Follow-Up Studies

**Figure 4:**
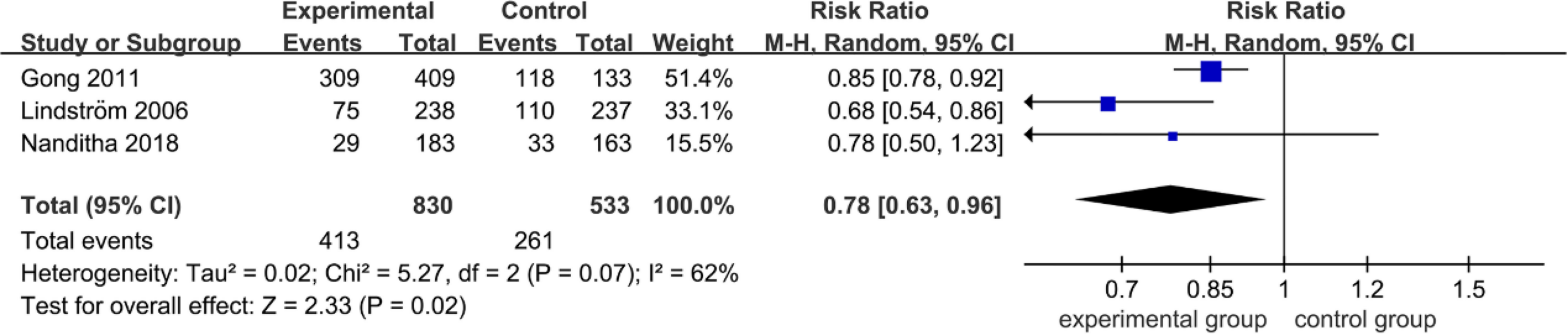
Forest plot of diabetes incidence in extended follow-up studies.

The three articles (Gong et al.; Nanditha et al.; Lindström et al.) were reported with diabetes outcomes of extended follow-up of intervention completed. As **Figure 4** shows, the overall diabetes incidence was clearly reduction (0.78; 95% CI 0.63 to 0.96, *p* = 0.07), with evidence of substantial heterogeneity (*I* ^2^ = 62%, *p* = 0.0). It was indicated that the past non-pharmacological interventions still had a long-term legacy effect to lower diabetes incidence. However, the heterogeneity across articles was moderate with an *I* ^2^ of 60%. In sensitivity analysis, the heterogeneity of combined estimates did mark a change with the exclusion of Gong et al. or Lindström et al. (**Table 3**), indicating that the source of heterogeneity may be due to the different participants (participants of Gong et al. and Lindström et al. were prediabetes, Nanditha et al. were diabetes).

**Table 3:** The sensitivity analysis outcomes of diabetes incidence in extended follow-up studies.

Removal of study	Intervention, N	Control, N	Pooled effect estimate			Heterogeneity	
			RR	95% Cl	p-value	I^2^	p_Z_
None	0	0	0.78	[0.63, 0.96]	0.07	62%	0.02
Gong, 2011 (22)	309/409	118/133	0.70	[0.57, 0.86]	0.58	0%	0.0007
Lindström, 2006 (25)	75/238	110/237	0.85	[0.78, 0.92]	0.66	0%	<0.0001
Nanditha, 2018 (24)	29/183	33/163	0.78	[0.59, 1.02]	0.02	81%	0.07

### Meta-Regression and Subgroup Analysis

**Figure 5:**
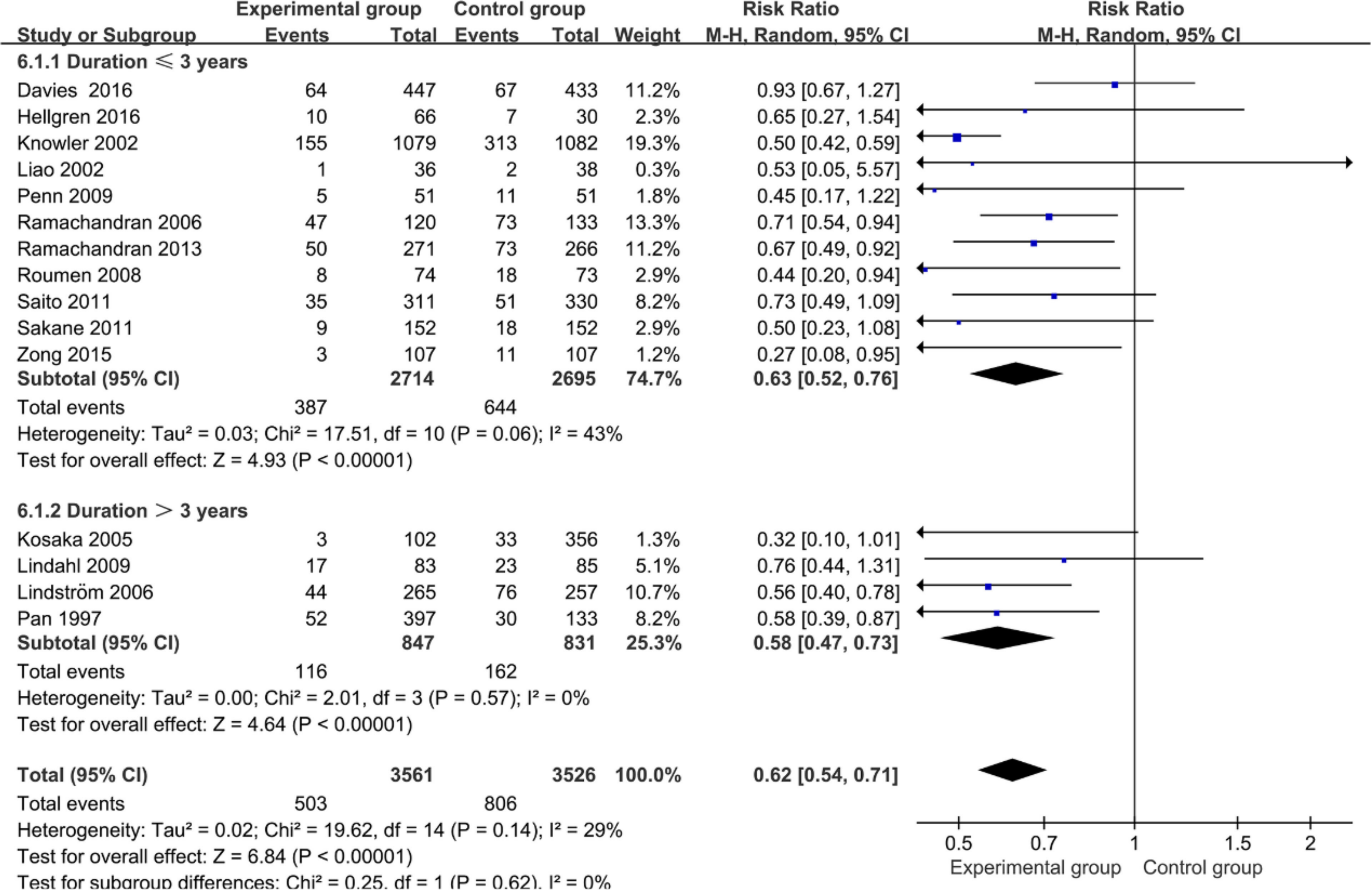
Forest plot of subgroup analysis of diabetes incidence in intervention studies.

Of the 21 articles, the 17 articles (the length of duration ranged from 2 to 6 years) were used to analyze the effect on diabetes prevention in intervention studies. To further evaluate the impact of duration on diabetes incidence, a random-effect meta-regression was conducted. The results of meta-regression analysis showed that duration (coefficient: -0.312, 95% CI: -0.457 to 0.166. p: 0.000) had a significant effect on prevention of diabetes. Based on the results of the meta-regression analysis, the subgroup analysis was performed for diabetes outcomes by follow-up duration (≤3 years, >3 years). As **Figure 5** shows, the overall diabetes incidence was clearly reduced (0.62; 95% CI 0.54 to 0.71, *p*<0.01), with evidence of low heterogeneity (*I^2^* = 29%, *p* = 0.14). Moreover, the diabetes incidence of the long-term duration group was clearly reduced by 0.05% compared with the relatively short-term-duration group.

### Publication Bias

Publication bias was assessed for diabetes outcome of 15 intervention studies. Details of quality of bias assessment of the included studies are listed in **Table 4**, and the funnel plot of 15 articles is provided in **Supplementary Table S4**.

**Table 4:** Quality of bias assessment of the included studies according to Cochrane guidelines.

First author (year)	Sequence generation	Allocation concealment	Blinding of participants, personnel, and outcome	Incomplete outcome data	Selective outcome reporting	Other potential threats to validity
Pan, 1997 (21)	L	L	H	L	L	L
Gong, 2011 (22)	L	L	H	L	L	L
Ramachandran, 2013 (23)	L	L	H	L	L	L
Nanditha, 2018 (24)	L	L	H	L	L	L
Lindström, 2006 (25)	L	L	H	L	L	L
Aro, 2019 (14)	L	L	H	L	L	L
Knowler, 2002 (26)	L	L	L	L	L	L
The Diabetes Prevention Program Research Group, 2015 (27)	L	L	H	L	L	L
Penn, 2009 (28)	L	L	H	L	L	L
Roumen, 2008 (39)	L	L	H	L	L	L
Davies, 2016 (29)	L	L	H	L	L	L
Balducci, 2006 (30)	L	L	H	L	L	L
Kosaka, 2005 (31)	L	L	H	L	L	L
Ramachandran, 2006 (32)	L	L	H	L	L	L
Liao, 2002 (33)	L	L	H	L	L	L
Lindahl, 2009 (34)	L	L	H	L	L	L
Saito, 2011 (35)	L	L	H	L	L	L
Sakane, 2011 (36)	L	L	H	L	L	L
Zong, 2015 (37)	L	L	U	L	L	L
Hellgren, 2016 (38)	L	L	U	L	L	L

L, low risk of bias; H, high risk of bias; U, unclear risk of bias.

## Discussion

In this systematic review and meta-analysis of 16 RCTs comparing non-pharmacological interventions with usual care, standard care, and medical therapy in prediabetes and diabetes, use of non-pharmacological interventions led to a long-term effect on reduction in the overall diabetes incidence and microvascular complications. Our forest plot of meta-analysis and sensitivity analysis demonstrated clearly that non-pharmacological interventions have such a reliable and long-term effect on the reduction in the microvascular complications among patients with hyperglycemia.

Our findings are consistent with several previous meta-analysis results which also indicated that the comprehensive non-pharmacological interventions showed significant effects on prevention of diabetes incidence and its risk of complications. Among them, lifestyle modification accounted for a large proportion, and previous meta-analysis studies have demonstrated a significant impact of lifestyle intervention on reduction of diabetes incidence and diabetes-related complications, as well as benefit in risk factors of cardiovascular disease (40–43). Moreover, several articles of meta-analysis show that physical activity and diet modification are also associated with a decrease in blood glucose and diabetes incidence (17, 44, 45). In addition, international guidelines recommended the year rate of diabetes incidence in diabetes prevention studies. In our meta-analysis, we have included that the follow-up duration of these studies was more than 2 years and even 20 years, and we not only analyzed the long-term effects of non-pharmacological therapies on diabetes incidence but also analyzed the efficacy of preventing microvascular events. Importantly, different intensities of intervention may affect the outcomes among participants. Low intensity of intervention may change the outcomes weakening the effects of interventions in these patients. However, with the increase of age, the intensity of intervention strategies and patients’ compliance may weaken as time goes by. We aimed to evaluate the relationship of un-pharmacotherapy with pharmacotherapy or placebo, and we did not evaluate the effects between different intensities of lifestyle intervention or physical activity in our analysis. The results of this pooled analysis show that non-pharmacological therapies (lifestyle intervention, physical activity, exercise, etc.) have a significant effect on the reduction in diabetes incidence in intervention studies and extended follow-up studies among prediabetic patients, and protection of diabetes from microvascular diseases. It is indicated that even a low intensity of intervention could lead to a significant effect and past interventions have had a very long-term effect and metabolic memory.

Some previous meta-analyses have reported relating outcomes of diabetic complications, such as the risk of cardiovascular events, and fewer systematic reviews have reported the chronic complications of diabetes in non-pharmacological studies. Since there are fewer studies that reported cardiovascular events in non-pharmacological studies, we have only analyzed the results of microvascular events. Originally, we have included RCTs of non-pharmacological-interventions in traditional Chinese medicine and bariatric surgeries. Compared with the medical therapies, bariatric and metabolic surgeries have been shown to be effective at preventing microvascular complications among patients with obesity and T2DM (46, 47). Bariatric and metabolic surgeries are not long-term interventions. Therefore, we updated the inclusion criteria and did not include surgery-related studies. In addition, bariatric and metabolic surgeries were invasive therapies through gastrectomy. Its necessity, safety, and postoperative complications should be carefully considered. Moreover, durations of non-pharmacological interventions of traditional Chinese medicine in diabetes prevention studies, such as acupuncture and Qigong, have been reported for less than 1 year, so we exclude them.

The strengths of this study include that it is a comprehensive non-pharmacological intervention (included RCTs more than 2 years and extended follow-up studies) that evaluates the effects on diabetes incidence and microvascular events. It was indicated that the past non-pharmacological interventions have had a long-term metabolic memory to prevent diabetes and its complications. This study has several limitations. First, regarding intervention strategies, only four intervention measures (lifestyle intervention, exercise, diet modification, and physical activity) met the inclusion criteria of this meta-analysis. Second is the variation in follow-up among the studies, ranging from 2 to 20 years. Last, due to the different treatment courses and different populations of patients, the heterogeneity remains relatively high among studies that evaluate the effect of reduction of diabetes incidence and microvascular complications.

## Conclusion

Overall, non-pharmacological strategies implemented are promising approaches for preventing diabetes among prediabetic patients and microvascular complications among patients with hyperglycemia, with a long-term significant effect. More prospective randomized clinical trials and extended follow-up are needed to evaluate the non-pharmacological strategies on diabetes and microvascular events.

## Data Availability Statement

The original contributions presented in the study are included in the article/**Supplementary Material**. Further inquiries can be directed to the corresponding author.

## Author Contributions

FL conceived and designed the analysis. RZ and YC extracted the data and performed the analysis. RZ, YC, and YhZ wrote the paper and made the figures and tables. JD, XA, YD, YqZ, and XK provided the critical revision of the manuscript. All authors contributed to the article and approved the submitted version.

## Funding

This work was funded by the 2015 Traditional Chinese Medicine Scientific Research (No. 201507001-11).

## Conflict of Interest

The authors declare that the research was conducted in the absence of any commercial or financial relationships that could be construed as a potential conflict of interest.

## Publisher’s Note

All claims expressed in this article are solely those of the authors and do not necessarily represent those of their affiliated organizations, or those of the publisher, the editors and the reviewers. Any product that may be evaluated in this article, or claim that may be made by its manufacturer, is not guaranteed or endorsed by the publisher.
