# Supplementary material for: The Long-Term Effects of Non-Pharmacological Interventions on Diabetes and Chronic Complication Outcomes in Patients With Hyperglycemia: A Systematic Review and Meta-Analysis
Source: Front Endocrinol (Lausanne). 2022 Mar 18;13:838224. doi: 10.3389/fendo.2022.838224 (PMC8971720; doi:10.3389/fendo.2022.838224)

Table S1 The PRISM checklist of this meta-analysis.

| **Section and Topic** | **Item #** | **Checklist item** | **Location where item is reported** |
| --- | --- | --- | --- |
| **TITLE** | | |  |
| Title | 1 | Identify the report as a systematic review. | 1 |
| **ABSTRACT** | | |  |
| Abstract | 2 | See the PRISMA 2020 for Abstracts checklist. | 1-2 |
| **INTRODUCTION** | | |  |
| Rationale | 3 | Describe the rationale for the review in the context of existing knowledge. | 2 |
| Objectives | 4 | Provide an explicit statement of the objective(s) or question(s) the review addresses. | 2 |
| **METHODS** | | |  |
| Eligibility criteria | 5 | Specify the inclusion and exclusion criteria for the review and how studies were grouped for the syntheses. | 3 |
| Information sources | 6 | Specify all databases, registers, websites, organisations, reference lists and other sources searched or consulted to identify studies. Specify the date when each source was last searched or consulted. | 3 |
| Search strategy | 7 | Present the full search strategies for all databases, registers and websites, including any filters and limits used. | 3 |
| Selection process | 8 | Specify the methods used to decide whether a study met the inclusion criteria of the review, including how many reviewers screened each record and each report retrieved, whether they worked independently, and if applicable, details of automation tools used in the process. | 3 |
| Data collection process | 9 | Specify the methods used to collect data from reports, including how many reviewers collected data from each report, whether they worked independently, any processes for obtaining or confirming data from study investigators, and if applicable, details of automation tools used in the process. | 3 |
| Data items | 10a | List and define all outcomes for which data were sought. Specify whether all results that were compatible with each outcome domain in each study were sought (e.g. for all measures, time points, analyses), and if not, the methods used to decide which results to collect. | 3 |
|  | 10b | List and define all other variables for which data were sought (e.g. participant and intervention characteristics, funding sources). Describe any assumptions made about any missing or unclear information. | 3 |
| Study risk of bias assessment | 11 | Specify the methods used to assess risk of bias in the included studies, including details of the tool(s) used, how many reviewers assessed each study and whether they worked independently, and if applicable, details of automation tools used in the process. | 4 |
| Effect measures | 12 | Specify for each outcome the effect measure(s) (e.g. risk ratio, mean difference) used in the synthesis or presentation of results. | 4 |
| Synthesis methods | 13a | Describe the processes used to decide which studies were eligible for each synthesis (e.g. tabulating the study intervention characteristics and comparing against the planned groups for each synthesis (item #5)). | 4 |
|  | 13b | Describe any methods required to prepare the data for presentation or synthesis, such as handling of missing summary statistics, or data conversions. | 4 |
|  | 13c | Describe any methods used to tabulate or visually display results of individual studies and syntheses. | 4 |
|  | 13d | Describe any methods used to synthesize results and provide a rationale for the choice(s). If meta-analysis was performed, describe the model(s), method(s) to identify the presence and extent of statistical heterogeneity, and software package(s) used. | 4 |
|  | 13e | Describe any methods used to explore possible causes of heterogeneity among study results (e.g. subgroup analysis, meta-regression). | 4 |
|  | 13f | Describe any sensitivity analyses conducted to assess robustness of the synthesized results. | 4 |
| Reporting bias assessment | 14 | Describe any methods used to assess risk of bias due to missing results in a synthesis (arising from reporting biases). | 4 |
| Certainty assessment | 15 | Describe any methods used to assess certainty (or confidence) in the body of evidence for an outcome. | 4 |
| **RESULTS** | | |  |
| Study selection | 16a | Describe the results of the search and selection process, from the number of records identified in the search to the number of studies included in the review, ideally using a flow diagram. | 4-5 |
|  | 16b | Cite studies that might appear to meet the inclusion criteria, but which were excluded, and explain why they were excluded. | 5-6 |
| Study characteristics | 17 | Cite each included study and present its characteristics. | 5-6 |
| Risk of bias in studies | 18 | Present assessments of risk of bias for each included study. | 7 |
| Results of individual studies | 19 | For all outcomes, present, for each study: (a) summary statistics for each group (where appropriate) and (b) an effect estimate and its precision (e.g. confidence/credible interval), ideally using structured tables or plots. | 6 |
| Results of syntheses | 20a | For each synthesis, briefly summarise the characteristics and risk of bias among contributing studies. | 6-7 |
|  | 20b | Present results of all statistical syntheses conducted. If meta-analysis was done, present for each the summary estimate and its precision (e.g. confidence/credible interval) and measures of statistical heterogeneity. If comparing groups, describe the direction of the effect. | 6-7 |
|  | 20c | Present results of all investigations of possible causes of heterogeneity among study results. | 6-7 |
|  | 20d | Present results of all sensitivity analyses conducted to assess the robustness of the synthesized results. | 6-7 |
| Reporting biases | 21 | Present assessments of risk of bias due to missing results (arising from reporting biases) for each synthesis assessed. | 6-7 |
| Certainty of evidence | 22 | Present assessments of certainty (or confidence) in the body of evidence for each outcome assessed. | 7 |
| **DISCUSSION** | | |  |
| Discussion | 23a | Provide a general interpretation of the results in the context of other evidence. | 8 |
|  | 23b | Discuss any limitations of the evidence included in the review. | 8-9 |
|  | 23c | Discuss any limitations of the review processes used. | 9 |
|  | 23d | Discuss implications of the results for practice, policy, and future research. | 9 |
| **OTHER INFORMATION** | | |  |
| Registration and protocol | 24a | Provide registration information for the review, including register name and registration number, or state that the review was not registered. | 2-3 |
|  | 24b | Indicate where the review protocol can be accessed, or state that a protocol was not prepared. | 2-3 |
|  | 24c | Describe and explain any amendments to information provided at registration or in the protocol. | 2-3 |
| Support | 25 | Describe sources of financial or non-financial support for the review, and the role of the funders or sponsors in the review. | 9 |
| Competing interests | 26 | Declare any competing interests of review authors. | 9 |
| Availability of data, code and other materials | 27 | Report which of the following are publicly available and where they can be found: template data collection forms; data extracted from included studies; data used for all analyses; analytic code; any other materials used in the review. | 9-10 |

Table S2 The search strategy of this meta-analysis.

| PubMed | Embase | Cochrane | Web of Science | MEDLINE |
| --- | --- | --- | --- | --- |
| 1. diabetes[MESH]  2. diabet*[Title/Abstract]  3. 1 or 2  4. lifestyle[MESH]  5. life style[Title/Abstract]  6. exercise[Title/Abstract]  7. diet*[Title/Abstract]  8. education[Title/Abstract]  9. physical activity[Title/Abstract]  10. behaviour[Title/Abstract]  11. nutrition[Title/Abstract]  12. nurture[Title/Abstract]  13. or/4-12  14. diabetic nephropathy[MESH]  15. Nephropat*[Title/Abstract]  16. Kidney[Title/Abstract]  17. renal[Title/Abstract]  18. Microvascular complications[Title/Abstract]  19. or/14-18  20. Diabetic retinopathy[MESH]  21. Retinopath*[Title/Abstract]  22. Retinal abnormality [Title/Abstract]  23. macular edema[Title/Abstract]  24. or/20-23  25. Diabetic peripheral neuropathy[MESH]  26. Neuropath*[Title/Abstract]  27. Neuralgia*[Title/Abstract]  28. or/25-27  29. cardiovascular disease[MESH]  30. coronary disease[Title/Abstract]  31. cardiomyopath*[Title/Abstract]  32. death[Title/Abstract]  33. Macrovascular complications[Title/Abstract]  34. or/29-33  35. 19 or 24 or 28 or 34  36.prediabetic state[MESH]  37. prediabet*[Title/Abstract]  38. Impaired glucose tolerance[Title/Abstract]  39. IGT[Title/Abstract]  40. Impaired fasting glucose[Title/Abstract]  41. IFG[Title/Abstract]  42. or/36-41  43. diabetes incidence[Title/Abstract]  44. 35 or 43  45. 3 and 13 and 35  46. 13 and 42 and 44  47. or/45-46  48. Randomized controlled trial[Filter] | 1. diabet*.mp.  2. lifestyle.mp.  3. life style.mp.  4. exercise.mp.  5. diet*.mp.  6. education.mp.  7. physical activity.mp.  8. behaviour.mp.  9. nutrition.mp.  10. nurture.mp.  11. or/2-10  12. (diabetic adj3 nephropathy).mp.  13. Nephropat*.mp.  14. Kidney.mp.  15. renal.mp.  16. Microvascular complications.mp.  17. (diabetic adj3 retinopathy).mp.  18. Retinopath*. mp.  19. (Retinal adj3 abnormality).mp.  20. (macular adj3 edema)  21. (Diabetic adj3 peripheral adj3 neuropathy).mp.  22. Neuropath*.mp.  23. Neuralgia*. mp.  24. (cardiovascular adj3 disease).mp.  25. (coronary adj3 disease). mp.  26. cardiomyopath*  27. death. mp.  28. (Macrovascular adj3 complications). mp.  29. or/12-28  30. prediabet*. mp.  31. (Impaired adj3 glucose adj3 tolerance).mp.  32. IGT. mp.  33. (Impaired adj3 fasting adj3 glucose). mp.  34. IFG. mp.  35. or/30-34  36. (randomized adj3 controlled adj3 trial).mp.  37. (randomized- controlled adj3 trial).mp.  38. randomly.mp.  39. or/36-38  40. (diabetes adj3 incidence).mp.  41.29 or 40  42.1 and 11 and 29  43.11 and 35 and 41  44. or/42-43  45. 44 and 39 | 1. diabet*.ti,ab,hw.  2. lifestyle.ti,ab,hw.  3. life style.ti,ab,hw.  4. exercise. ti,ab,hw.  5. diet*.ti,ab,hw.  6. education.ti,ab,hw.  7. physical activity.ti,ab,hw.  8. behaviour.ti,ab,hw.  9. nutrition.ti,ab,hw.  10. nurture.ti,ab,hw.  11. or/2-10  12. (diabetic adj3 nephropathy).ti,ab,hw.  13. Nephropat*.ti,ab,hw.  14. Kidney.ti,ab,hw.  15. renal.ti,ab,hw.  16. Microvascular complications.ti,ab,hw.  17. (diabetic adj3 retinopathy).ti,ab,hw.  18. Retinopath*.ti,ab,hw.  19. (Retinal adj3 abnormality).ti,ab,hw.  20. (macular adj3 edema).ti,ab,hw.  21. (Diabetic adj3 peripheral adj3 neuropathy).ti,ab,hw.  22. Neuropath*.ti,ab,hw.  23. Neuralgia*.ti,ab,hw.  24. (cardiovascular adj3 disease).ti,ab,hw.  25. (coronary adj3 disease). ti,ab,hw.  26. cardiomyopath* ti,ab,hw.  27. death. ti,ab,hw.  28. (Macrovascular adj3 complications). ti,ab,hw.  29. or/12-28  30. prediabet*. ti,ab,hw.  31. (Impaired adj3 glucose adj3 tolerance). ti,ab,hw.  32. IGT. ti,ab,hw.  33. (Impaired adj3 fasting adj3 glucose). ti,ab,hw.  34. IFG. ti,ab,hw.  35. or/30-34  36. (randomized adj3 controlled adj3 trial). ti,ab,hw.  37. (randomized- controlled adj3 trial). ti,ab,hw.  38. randomly. ti,ab,hw.  39. or/36-38  40. (diabetes adj3 incidence). ti,ab,hw.  41.29 or 40  42.1 and 11 and 29  43.11 and 35 and 41  44. or/42-43  45. 44 and 39 | 1. diabet*.mp.  2. lifestyle.mp.  3. life style.mp.  4. exercise.mp.  5. diet*.mp.  6. education.mp.  7. physical activity.mp.  8. behaviour.mp.  9. nutrition.mp.  10. nurture.mp.  11. or/2-10  12. (diabetic adj3 nephropathy).mp.  13. Nephropat*.mp.  14. Kidney.mp.  15. renal.mp.  16. Microvascular complications.mp.  17. (diabetic adj3 retinopathy).mp.  18. Retinopath*. mp.  19. (Retinal adj3 abnormality). mp.  20. (macular adj3 edema)  21. (Diabetic adj3 peripheral adj3 neuropathy).mp.  22. Neuropath*.mp.  23. Neuralgia*. mp.  24. (cardiovascular adj3 disease). mp.  25. (coronary adj3 disease).mp.  26. cardiomyopath*  27. death. mp.  28. (Macrovascular adj3 complications). mp.  29. or/12-28  30. prediabet*. mp.  31. (Impaired adj3 glucose adj3 tolerance). mp.  32. IGT. mp.  33. (Impaired adj3 fasting adj3 glucose). mp.  34. IFG. mp.  35. or/30-34  36. (randomized adj3 controlled adj3 trial). mp.  37. (randomized- controlled adj3 trial).mp.  38. randomly.mp.  39. or/36-38  40. (diabetes adj3 incidence).mp.  41.29 or 40  42.1 and 11 and 29  43.11 and 35 and 41  44. or/42-43  45. 44 and 39 | 1. diabet*.mp.  2. lifestyle.mp.  3. life style.mp.  4. exercise.mp.  5. diet*.mp.  6. education.mp.  7. physical activity.mp.  8. behaviour.mp.  9. nutrition.mp.  10. nurture.mp.  11. or/2-10  12. (diabetic adj3 nephropathy).mp.  13. Nephropat*.mp.  14. Kidney.mp.  15. renal.mp.  16. Microvascular complications.mp.  17. (diabetic adj3 retinopathy).mp.  18. Retinopath*. mp.  19. (Retinal adj3 abnormality).mp.  20. (macular adj3 edema)  21. (Diabetic adj3 peripheral adj3 neuropathy).mp.  22. Neuropath*.mp.  23. Neuralgia*. mp.  24. (cardiovascular adj3 disease).mp.  25. (coronary adj3 disease). mp.  26. cardiomyopath*  27. death. mp.  28. (Macrovascular adj3 complications). mp.  29. or/12-28  30. prediabet*. mp.  31. (Impaired adj3 glucose adj3 tolerance). mp.  32. IGT. mp.  33. (Impaired adj3 fasting adj3 glucose). mp.  34. IFG. mp.  35. or/30-34  36. (randomized adj3 controlled adj3 trial). mp.  37. (randomized- controlled adj3 trial).mp.  38. randomly.mp.  39. or/36-38  40. (diabetes adj3 incidence).mp.  41.29 or 40  42.1 and 11 and 29  43.11 and 35 and 41  44. or/42-43  45. 44 and 39 |

Note: The initial search was performed on March 2021, and an updated search of five databases was performed on November 2021 using the same search terms.

Table S3. Trials excluded from meta-analysis

| Balk-Møller NC, Poulsen SK, Larsen TM. Effect of a Nine-Month Web- and App-Based Workplace Intervention to Promote Healthy Lifestyle and Weight Loss for Employees in the Social Welfare and Health Care Sector: A Randomized Controlled Trial. J Med Internet Res 2017;19(4):e108. doi: 10.2196/jmir.6196 | studies duration less than 2 years |
| --- | --- |
| Elmer PJ, Obarzanek E, Vollmer WM, et al. Effects of comprehensive lifestyle modification on diet, weight, physical fitness, and blood pressure control: 18-month results of a randomized trial. Ann Intern Med 2006;144(7):485-95. doi: 10.7326/0003-4819-144-7-200604040-00007 | studies duration less than 2 years |
| Wani K, Alfawaz H, Alnaami AM, et al. Effects of A 12-Month Intensive Lifestyle Monitoring Program in Predominantly Overweight/Obese Arab Adults with Prediabetes. Nutrients 2020;12(2) doi: 10.3390/nu12020464 | studies duration less than 2 years |
| Yamauchi K, Katayama T, Yamauchi T, et al. Efficacy of a 3-month lifestyle intervention program using a Japanese-style healthy plate on body weight in overweight and obese diabetic Japanese subjects: a randomized controlled trial. Nutr J 2014;13:108. doi: 10.1186/1475-2891-13-108 | studies duration less than 2 years |
| Manios Y, Lambrinou CP, Mavrogianni C, et al. Lifestyle Changes Observed among Adults Participating in a Family- and Community-Based Intervention for Diabetes Prevention in Europe: The 1(st) Year Results of the Feel4Diabetes-Study. Nutrients 2020;12(7) doi: 10.3390/nu12071949 | studies duration less than 2 years |
| Pedrosa C, Oliveira BM, Albuquerque I, et al. Metabolic syndrome, adipokines and ghrelin in overweight and obese schoolchildren: results of a 1-year lifestyle intervention programme. Eur J Pediatr 2011;170(4):483-92. doi: 10.1007/s00431-010-1316-2 | studies duration less than 2 years |
|  | studies duration less than 2 years |
| Hansen LJ, Siersma V, Beck-Nielsen H, et al. Structured personal care of type 2 diabetes: a 19 year follow-up of the study Diabetes Care in General Practice (DCGP). Diabetologia 2013;56(6):1243-53. doi: 10.1007/s00125-013-2893-1 | compared 2 treatments of nonpharmacological interventions |
| Díaz-López A, Babio N, Martínez-González MA, et al. Mediterranean Diet, Retinopathy, Nephropathy, and Microvascular Diabetes Complications: A Post Hoc Analysis of a Randomized Trial. Diabetes Care 2015;38(11):2134-41. doi: 10.2337/dc15-1117 | compared 2 treatments of nonpharmacological interventions |
| Wing RR, Bolin P, Brancati FL, et al. Cardiovascular effects of intensive lifestyle intervention in type 2 diabetes. N Engl J Med 2013;369(2):145-54. doi: 10.1056/NEJMoa1212914 | compared 2 treatments of nonpharmacological interventions |
| Effects of a long-term lifestyle modification programme on peripheral neuropathy in overweight or obese adults with type 2 diabetes: the Look AHEAD study. Diabetologia 2017;60(6):980-88. doi: 10.1007/s00125-017-4253-z | compared 2 treatments of nonpharmacological interventions |
| Olsen RH, Pedersen LR, Jürs A, et al. A randomised trial comparing the effect of exercise training and weight loss on microvascular function in coronary artery disease. Int J Cardiol 2015;185:229-35. doi: 10.1016/j.ijcard.2015.03.118 | unsuitable endpoints |
| Adverse events and their association with treatment regimens in the diabetes control and complications trial. Diabetes Care 1995;18(11):1415-27. doi: 10.2337/diacare.18.11.1415 | unsuitable endpoints |
| Schauer PR, Bhatt DL, Kirwan JP, et al. Bariatric Surgery versus Intensive Medical Therapy for Diabetes - 5-Year Outcomes. N Engl J Med 2017;376(7):641-51. doi: 10.1056/NEJMoa1600869 | unsuitable endpoints |
| Mingrone G, Panunzi S, De Gaetano A, et al. Bariatric-metabolic surgery versus conventional medical treatment in obese patients with type 2 diabetes: 5 year follow-up of an open-label, single-centre, randomised controlled trial. Lancet 2015;386(9997):964-73. doi: 10.1016/s0140-6736(15)00075-6 | unsuitable endpoints |
| Simonson DC, Halperin F, Foster K, et al. Clinical and Patient-Centered Outcomes in Obese Patients With Type 2 Diabetes 3 Years After Randomization to Roux-en-Y Gastric Bypass Surgery Versus Intensive Lifestyle Management: The SLIMM-T2D Study. Diabetes Care 2018;41(4):670-79. doi: 10.2337/dc17-0487 | unsuitable endpoints |
| Blackberry ID, Furler JS, Best JD, et al. Effectiveness of general practice based, practice nurse led telephone coaching on glycaemic control of type 2 diabetes: the Patient Engagement and Coaching for Health (PEACH) pragmatic cluster randomised controlled trial. Bmj 2013;347:f5272. doi: 10.1136/bmj.f5272 | unsuitable endpoints |
| Wing RR. Long-term effects of a lifestyle intervention on weight and cardiovascular risk factors in individuals with type 2 diabetes mellitus: four-year results of the Look AHEAD trial. Arch Intern Med 2010;170(17):1566-75. doi: 10.1001/archinternmed.2010.334 | unsuitable endpoints |
| Park DW, Kim YH, Song HG, et al. Long-term outcome of stents versus bypass surgery in diabetic and nondiabetic patients with multivessel or left main coronary artery disease: a pooled analysis of 5775 individual patient data. Circ Cardiovasc Interv 2012;5(4):467-75. doi: 10.1161/circinterventions.112.969915 | unsuitable endpoints |
| Shroyer ALW, Quin JA, Wagner TH, et al. Off-Pump Versus On-Pump Impact: Diabetic Patient 5-Year Coronary Artery Bypass Clinical Outcomes. Ann Thorac Surg 2019;107(1):92-98. doi: 10.1016/j.athoracsur.2018.07.076 | unsuitable endpoints |
| Ikramuddin S, Billington CJ, Lee WJ, et al. Roux-en-Y gastric bypass for diabetes (the Diabetes Surgery Study): 2-year outcomes of a 5-year, randomised, controlled trial. Lancet Diabetes Endocrinol 2015;3(6):413-22. doi: 10.1016/s2213-8587(15)00089-3 | unsuitable endpoints |
| Toobert DJ, Strycker LA, Barrera M, et al. Seven-year follow-up of a multiple-health-behavior diabetes intervention. Am J Health Behav 2010;34(6):680-94. doi: 10.5993/ajhb.34.6.5 | unsuitable endpoints |
| Bonaccio M, Di Castelnuovo A, Costanzo S, et al. Adherence to the traditional Mediterranean diet and mortality in subjects with diabetes. Prospective results from the MOLI-SANI study. Eur J Prev Cardiol 2016;23(4):400-7. doi: 10.1177/2047487315569409 | non-randomized study |
| Fisher DP, Johnson E, Haneuse S, et al. Association Between Bariatric Surgery and Macrovascular Disease Outcomes in Patients With Type 2 Diabetes and Severe Obesity. Jama 2018;320(15):1570-82. doi: 10.1001/jama.2018.14619 | non-randomized study |
| Sjöström L, Peltonen M, Jacobson P, et al. Association of bariatric surgery with long-term remission of type 2 diabetes and with microvascular and macrovascular complications. Jama 2014;311(22):2297-304. doi: 10.1001/jama.2014.5988 | non-randomized study |
| Aminian A, Zajichek A, Arterburn DE, et al. Association of Metabolic Surgery With Major Adverse Cardiovascular Outcomes in Patients With Type 2 Diabetes and Obesity. Jama 2019;322(13):1271-82. doi: 10.1001/jama.2019.14231 | non-randomized study |
| Strelitz J, Ahern AL, Long GH, et al. Changes in behaviors after diagnosis of type 2 diabetes and 10-year incidence of cardiovascular disease and mortality. Cardiovasc Diabetol 2019;18(1):98. doi: 10.1186/s12933-019-0902-5 | non-randomized study |
| Shepherd E, Gomersall JC, Tieu J, et al. Combined diet and exercise interventions for preventing gestational diabetes mellitus. Cochrane Database Syst Rev 2017;11(11):Cd010443. doi: 10.1002/14651858.CD010443.pub3 | non-randomized study |
| Eriksson KF, Lindgärde F. No excess 12-year mortality in men with impaired glucose tolerance who participated in the Malmö Preventive Trial with diet and exercise. Diabetologia 1998;41(9):1010-6. doi: 10.1007/s001250051024 | non-randomized study |
| Eriksson KF, Lindgärde F. Prevention of type 2 (non-insulin-dependent) diabetes mellitus by diet and physical exercise. The 6-year Malmö feasibility study. Diabetologia 1991;34(12):891-8. doi: 10.1007/bf00400196 | non-randomized study |
| Hanefeld M, Fischer S, Julius U, et al. Risk factors for myocardial infarction and death in newly detected NIDDM: the Diabetes Intervention Study, 11-year follow-up. Diabetologia 1996;39(12):1577-83. doi: 10.1007/s001250050617 | non-randomized study |
| Thankappan KR, Sathish T, Tapp RJ, et al. A peer-support lifestyle intervention for preventing type 2 diabetes in India: A cluster-randomized controlled trial of the Kerala Diabetes Prevention Program. PLoS Med 2018;15(6):e1002575. doi: 10.1371/journal.pmed.1002575 | without original data |
| Tan E, Khoo J, Gani LU, et al. Effect of multidisciplinary intensive targeted care in improving diabetes mellitus outcomes: a randomized controlled pilot study - the Integrated Diabetes Education, Awareness and Lifestyle modification in Singapore (IDEALS) Program. Trials 2019;20(1):549. doi: 10.1186/s13063-019-3601-3 | without original data |
| Tanaka S, Tanaka S, Iimuro S, et al. Cohort profile: The Japan diabetes complications study: a long-term follow-up of a randomised lifestyle intervention study of type 2 diabetes. Int J Epidemiol 2014;43(4):1054-62. doi: 10.1093/ije/dyt057 | unsuitable participants |
| Lau CJ, Pisinger C, Husemoen LLN, et al. Effect of general health screening and lifestyle counselling on incidence of diabetes in general population: Inter99 randomised trial. Prev Med 2016;91:172-79. doi: 10.1016/j.ypmed.2016.08.016 | unsuitable participants |
| Blomster JI, Chow CK, Zoungas S, et al. The influence of physical activity on vascular complications and mortality in patients with type 2 diabetes mellitus. Diabetes Obes Metab 2013;15(11):1008-12. doi: 10.1111/dom.12122 | unsuitable participants |
| Sone H, Tanaka S, Iimuro S, et al. Long-term lifestyle intervention lowers the incidence of stroke in Japanese patients with type 2 diabetes: a nationwide multicentre randomised controlled trial (the Japan Diabetes Complications Study). Diabetologia 2010;53(3):419-28. doi: 10.1007/s00125-009-1622-2 | unsuitable participants |
| Ponzo V, Gentile L, Gambino R, et al. Incidence of diabetes mellitus, cardiovascular outcomes and mortality after a 12-month lifestyle intervention: A 9-year follow-up. Diabetes Metab 2018;44(5):449-51. doi: 10.1016/j.diabet.2018.04.008 | unsuitable participants |
| Tolppanen AM, Pulkkinen L, Kolehmainen M, et al. Tenomodulin is associated with obesity and diabetes risk: the Finnish diabetes prevention study. Obesity (Silver Spring) 2007;15(5):1082-8. doi: 10.1038/oby.2007.613 | articles were from the same trial and without the outcome of interest |
| Uusitupa M, Peltonen M, Lindström J, et al. Ten-year mortality and cardiovascular morbidity in the Finnish Diabetes Prevention Study--secondary analysis of the randomized trial. PLoS One 2009;4(5):e5656. doi: 10.1371/journal.pone.0005656 | articles were from the same trial and without the outcome of interest |
| Uusitupa MI, Stancáková A, Peltonen M, et al. Impact of positive family history and genetic risk variants on the incidence of diabetes: the Finnish Diabetes Prevention Study. Diabetes Care 2011;34(2):418-23. doi: 10.2337/dc10-1013 | articles were from the same trial and without the outcome of interest |
| Apolzan JW, Venditti EM, Edelstein SL, et al. Long-Term Weight Loss With Metformin or Lifestyle Intervention in the Diabetes Prevention Program Outcomes Study. Ann Intern Med 2019;170(10):682-90. doi: 10.7326/m18-1605 | articles were from the same trial and without the outcome of interest |
| Li X, Wang J, Shen X, et al. Higher blood pressure predicts diabetes and enhances long-term risk of cardiovascular disease events in individuals with impaired glucose tolerance: Twenty-three-year follow-up of the Daqing diabetes prevention study. J Diabetes 2019;11(7):593-98. doi: 10.1111/1753-0407.12887 | articles were from the same trial and without the outcome of interest |
| Shen XX, Wang JP, Chen YY, et al. [Subjects with impaired glucose tolerance returned to normal glucose status for six years had lower long-term risk of diabetes: 20 years follow up of Daqing diabetes prevention study]. Zhonghua Nei Ke Za Zhi 2019;58(5):372-76. doi: 10.3760/cma.j.issn.0578-1426.2019.05.008 | articles were from the same trial and without the outcome of interest |

Table S4 The funnel plot of 15 articles of intervention studies


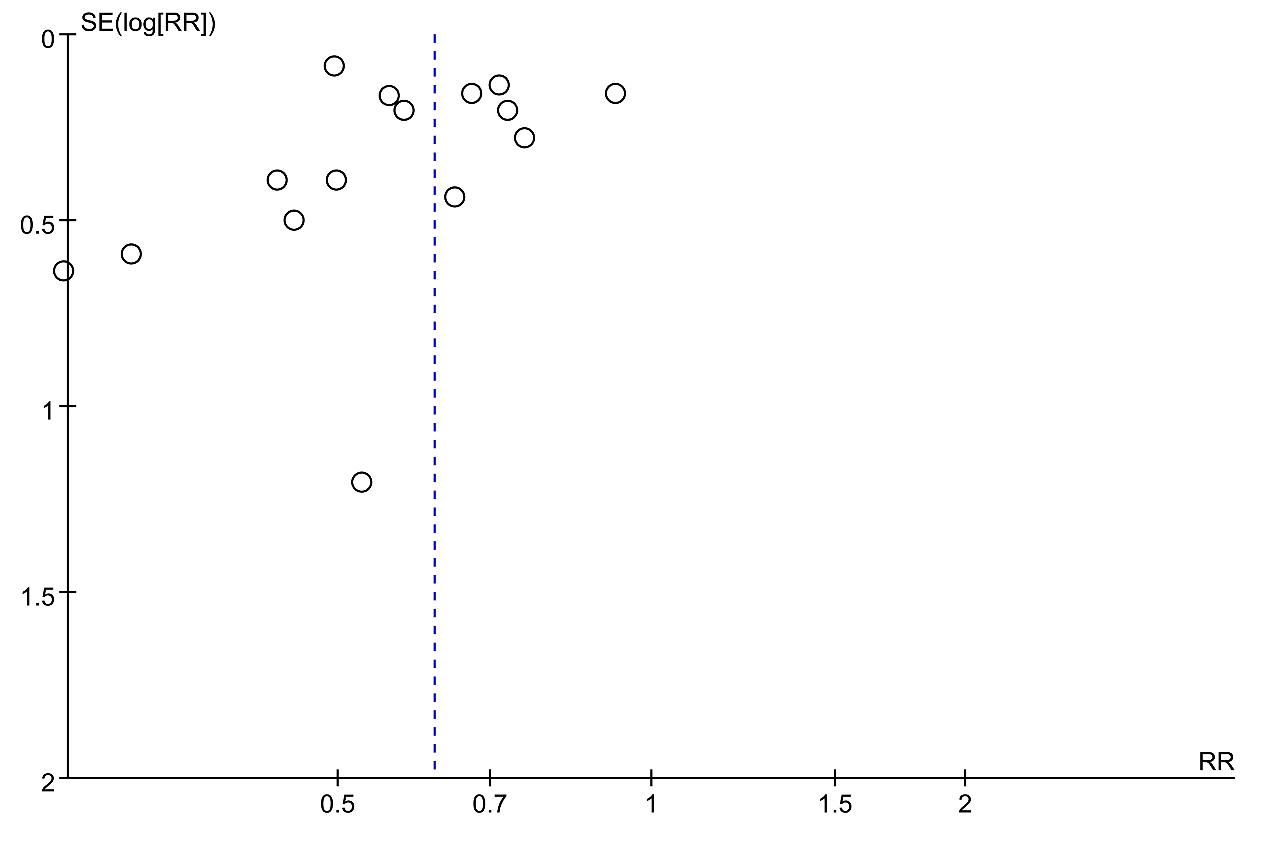

Supplement: Supplementary file 1 [file DataSheet_1.docx]
